# Effect of Dispersing Multiwalled Carbon Nanotubes and Graphene Nanoplatelets Hybrids in the Matrix on the Flexural Fatigue Properties of Carbon/Epoxy Composites

**DOI:** 10.3390/polym14050918

**Published:** 2022-02-25

**Authors:** Yi-Ming Jen, Wei-Lun Ni

**Affiliations:** Department of Mechanical and Mechatronic Engineering, National Taiwan Ocean University, Keelung City 202301, Taiwan; 10772012@mail.ntou.edu.tw

**Keywords:** multiwalled carbon nanotube (MWCNT), graphene nanoplatelet (GNP), carbon fiber reinforced epoxy laminate, flexural monotonic strength, flexural fatigue strength, synergistic effect, bridging effect, crack deflection effect

## Abstract

The synergistic effect of applying hybrid nanoparticles in improving the fatigue property of fiber reinforced polymer composites has rarely been explored before. Hence the monotonic and fatigue flexure properties of the carbon fiber reinforced epoxy laminates with matrix modified by multiwalled carbon nanotubes and graphene nanoplatelets were experimentally studied herein. The nanofiller ratio applied in the matrix modification was considered as a variable in the experimental program to investigate the effect of nanofiller ratio on the studied mechanical properties. A synergistic index has been employed to evaluate the synergistic effect of hybrid nanoparticles on the studied properties successfully. Experimental results show that the laminates with matrix modified under a nanofiller ratio (multiwalled carbon nanotube: graphene nanoplatelet) of 9:1 have the higher monotonic and fatigue strengths than those modified under other nanofiller ratios. The monotonic flexural strength and fatigue limit of the specimens modified under a nanofiller ratio of 9:1 are higher than the neat laminate specimens by 9.3 and 11.0%, respectively. The fatigue limits of the studied nano-modified laminates increase with the static strengths. Adding hybrid nanoparticles under proper nanofiller ratios in the matrix can suppress the degradation of the stiffness, further increase the resistance to fatigue damage. Examining the fracture surfaces of fatigued specimens reveals that the pullout/bridging effects of carbon nanotubes and the crack deflection effect of graphene nanoplatelets are the main reinforcement mechanisms in enhancing the fatigue strength of the composites.

## 1. Introduction

Owing to the characteristics of light weight, high specific strength, specific stiffness, and corrosion resistance, etc., fiber reinforced polymer (FRP) laminates have been widely applied in the aerospace, automobiles, shipbuilding, and construction industries. Accordingly, understanding the mechanical properties of the FRP composites subjected to various types of loading are critical to the application of these composite materials. In general, the FRP laminates with appropriately designed ply-sequences have excellent in-plane properties. However, the poor out-of-plane properties due to the fabrication of building-up laminates reduce the structural applicability of FRP composites significantly. Many efforts, such as three-dimensional weaving, embroidery, stitching and braiding [1,2,3,4], have been made to improve the interlaminar properties of the FRP laminates. Nevertheless, some in-plane strengths or properties may be lost due to the misalignment of fibers or ununiform distribution of resins when applying these techniques.

Recently, with the advancement of synthesis and modification technologies of nanoparticles, using nanoparticles to improve the mechanical properties of FRP laminates has been received much attention. Among the employed reinforcements, the carbon nanotubes (CNTs) and graphene-family particles are two commonly employed nanoparticles due to their characteristic one-dimensional and two-dimensional morphologies. Past studies regarding the reinforcement effect of these carbon nanoparticles on the mechanical properties of FRPs under quasi-static loading, such as tensile or flexural properties and interlaminar fracture toughness, have been reviewed in some references [5,6,7,8,9,10,11,12]. Although FRPs are often subjected to fluctuating loading in practical applications, unfortunately, the past investigations regarding the fatigue properties of the nanoparticle modified FRP laminates are relatively rare.

In general, past fatigue studies of FRP laminates can be divided into two categories according to the types of specimens used in the tests. One type of studies explored the relationship between the applied cyclic loads and the fatigue lives of the FRP composites. The bulk specimens were prepared and used in the fatigue tests to understand the correlation between the load and fatigue life. The other type of research employed the pre-cracked specimens to investigate the relationship between the fracture mechanics parameter ahead the crack fronts and the delamination growth rates. Surveying the limited past studies on the fatigue behavior of nanoparticle reinforced FRPs demonstrating that adding CNTs in the matrix can improve the tensile or flexural fatigue strengths of the bulk FRP specimens [13,14,15,16,17,18,19,20,21] and retard the delamination growth rates [22,23,24] of cracked FRP laminates due to the pullout and bridging mechanisms. In 2008, the effect of adding CNTs in the matrix on the high-cycle fatigue behavior of glass fiber reinforced epoxy composites was studied by Grimmer and Dharan [13]. The adding of small amount of CNTs was found to increase the high-cycle fatigue life and suppress the cyclic hysteresis evolution significantly. The similar improvement on the fatigue strength of MWCNT modified glass/epoxy composites was also reported in [14], and the reinforcement mechanism was contributed by the increase in inter fiber fracture strength. In the same year, the impact and post-impact fatigue strengths of carbon/epoxy composites were found to be increased by adding MWCNTs in the matrix [15]. The enhanced fracture toughness by adding MWCNTs was considered as the reason for increased impact and post-impact properties. The fatigue behavior of MWCNT modified carbon/epoxy composites has been investigated by Knoll et al. [20]. The addition of 0.3 wt.% MWCNTs was found to has no effect on the static strength but increase the fatigue life of neat laminates remarkedly. By examining the damage in terms of the stiffness degradation, the MWCNTs retarded the damage development with the fatigue cycles effectively. Moreover, the enhanced effect of MWCNTs on the fatigue strength of the CFRP composites was also observed at low temperatures [17]. Some studies reported the addition of CNTs on the mechanical properties has no influence on the fatigue properties. Borrego et al. [19] reported that the carbon/epoxy laminates modified by 0.5 wt.% MWCNTs has similar fatigue strength with the neat laminates. However, adding 1.0 wt.% MWCNTs has adverse effect on the fatigue properties due to the agglomeration of nanoparticles. In general, the addition of CNTs can decrease the delamination growth rates of fiber reinforced laminates. Fenner and Daniel [23] found that the mode I fatigue crack propagation rate of woven carbon/epoxy laminates can decrease by factor of two by dispersing 0.5 wt.% CNTs in the matrix. The pull-out of CNTs and the fractal surface of nanotube-enhanced material were employed to explain the reinforcement mechanisms.

For the graphene-family nanoparticles, the past works showed that the crack bifurcation and crack deflection effect improved the fatigue resistance of the bulk FRP specimens [20,21,25,26,27] and suppressed the delamination propagation rate of the pre-cracked laminates [28] when the crack fronts encountered the two-dimensional flake-shaped nanofillers. In 2010, Yavari et al. [25] found that the fatigue strength of GNP modified glass/epoxy composites increased with the amounts of GNPs up to 2.0 wt.%. The reinforcement of GNPs on the tensile and flexural fatigue strengths are higher than that of CNTs due to the graphene toughening networks. Similar results were reported in [20] that the few layered graphene (FLG) had higher enhanced effects on fatigue strength of carbon/epoxy laminates than CNTs. Shen et al. [26] found that the addition of 0.25 wt.% GNPs in the matrix can increase the fatigue life of the neat carbon/epoxy composites. However, opposite results were observed on the antisymmetric glass/epoxy laminates in [21]. The glass/epoxy composites modified by 0.1 wt.% SWCNTs were found to have higher fatigue strength than those modified by 0.1 wt.% GNPs. In 2020, Tareq et al. [28] proposed that the enhanced interlaminar fractur toughness resulting from the crack deflection effect of GNPs was the main reinforcement mechanism on fatigue strength of the carbon/epoxy composites. 

Recently, hybrid nanoparticle systems have been applied to improve the mechanical properties of polymer nanocomposites by combining the characteristics of two types of nanoparticles with different morphologies [29,30,31,32,33,34,35]. In 2020, Kumar et al. [34] reviewed the synergistic effects of CNTs and graphene hybrids on the properties of polymers and summarized that CNTs can increase the interlayer space of graphene and improve the load transfer, further enhance the mechanical properties of the polymers. The synergistic effect of the hybrid nanoparticles on the mechanical properties of polymers has been confirmed when the applied hybrid nanoparticles were under appropriate mixing ratios. However, the effect on hybrid nanoparticles on the improvement in fatigue strengths of FRP laminates has not been investigated till nowadays. Since most engineering materials are subjected to the fluctuating loading, the knowledge to extend the fatigue life of the innovative materials is needed urgently at the design and application stages. After the quasi-static strengths of FRPs has been improved using the nanofillers successfully, the application of hybrid nanofillers to retard the evolution of fatigue damage and increase fatigue strength of FRP laminates has become a topic worthy of study. Hence, the flexural monotonic and fatigue strengths of the carbon fiber reinforced epoxy (Cf/Ep) laminates with matrix modified by multiwalled CNTs (MWCNTs) and graphene nanoplatelets (GNPs) were experimentally explored in this study. The Cf/Ep laminate specimens with matrix modified under various hybrid-nanofiller ratios were prepared to investigate the effects of applied nanoparticle type and nanofiller ratios (MWCNT:GNP) on the flexural static and fatigue strengths of the Cf/Ep laminates. The fracture surfaces obtained after the flexural fatigue tests were also observed to verify the enhancement mechanisms of the MWCNT and GNP hybrids on the fatigue strengths of the studied laminates.

In the rest of this article, Section 2.1 reports the materials used in this study and Section 2.2 describes how epoxy resins are reinforced with nanofillers. The fabrication of Cf/Ep laminate specimens is introduced in Section 2.3 and the experimental methods for various mechanical properties are provided in Section 2.4. The results and related discussion of monotonic flexural tests and fatigue flexural tests on the nano-modified laminates Cf/Ep with different nanofiller ratios are described in Section 3.1 and Section 3.2, respectively. Section 3.3 illustrates the reinforcement mechanisms of nanofillers on the monotonic and fatigue properties by observing the fracture surfaces of the specimens. Some conclusions about this study are summarized in Section 4.

## 2. Materials and Methods

### 2.1. Materials

The specifications of the materials used in this study are listed in Table 1. The 12 k unidirectional carbon fabrics were used in the preparation of Cf/Ep laminates. The fabrics were fabricated by Formosa Taffeta Co., Ltd., Touliou, Taiwan, with the designation of ECCFM. The areal weight is 216 g/m^2^ and the thickness is 0.28 mm. The solvent-type epoxy resin employed in this study was supplied by Epotech Composite Co., Taichung, Taiwan, under the designation of EPO-622. The employed epoxy resin is composed of epoxy resin, dicyanamide curing agent, and methyl ethyl ketone (MEK) solvent. The weight ratio between the epoxy resin and curing agent is 18:2.

The MWCNTs utilized in this study was supplied from Nanocyl Ltd., Sambreville, Belgium, with the designation of NC7000. The diameter and length of the MWCNT are approximately 9.5 nm and 1.5 μm, respectively. The purity is higher than 90%. The employed GNPs was provided by Knano Co., Xiamen, China, with the designation of KNG-150. The diameter and the thickness of the as-received GNP are about 5 μm and 10 nm, respectively. The purity of the GNPs is higher than 99.5%. The as-received nanoparticles were chemically-modified using maleic anhydride (MA) to establish advantageous functional groups on the surface, which can provide favorable links between the nanoparticle and the polymer matrix. The functionalization of CNTs and graphene-family nanoparticles by using MA have been widely applied in past studies [36,37,38,39]. The employed MA modification of MWCNTs and GNPs in this study were performed according to the past studies [37]. According to the past analysis of Fourier transform infrared (FT-IR) spectroscopy on the MA modified MWCNT and GNP powder samples [37], the peaks appeared approximately at 1740 cm^−1^, corresponding to the C=O stretching vibration of carbonyl functional group. The functional group is a standard characteristics of acid anhydrides, which is easy to form binds on the surface of epoxy and further improves the dispersion of the nanofillers and enhances the crosslink between the nanofillers and the polymer matrix. 

### 2.2. Nano-Modified Epoxy Resins

**Figure 1:**
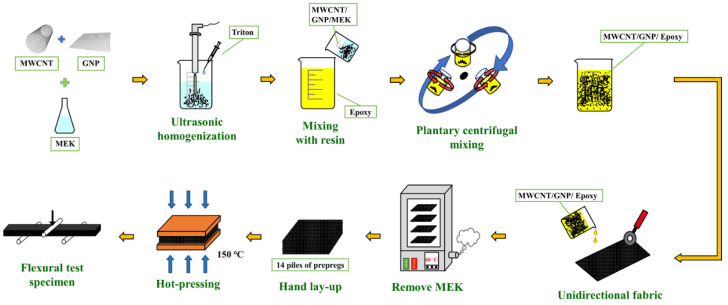
Schematic illustration of the procedure for specimen preparation.

The procedure of fabricating the specimens is schematically illustrated in Figure 1. The employed nanoparticles with the required weight were dried at 120 °C for 1 h to remove the moisture. Then the nanoparticles were added into the MEK solvent and dispersed using the ultrasonic homogenizer for 10 min. Next, the solution was added by the surfactant Triton X-405 (Sigma-Aldrich, Inc., St. Louis, MI, USA) and sonicated for another 20 min. Subsequently the suspension was mixed with epoxy resin and agitated using a planetary centrifuge mixer for 20 min to obtain the nano-modified epoxy resin. The concentration of the two nanofillers in the matrix was controlled at 0.5 wt.%.

### 2.3. Fabrication of Nano-Modified Cf/Ep Laminates 

**Figure 2:**
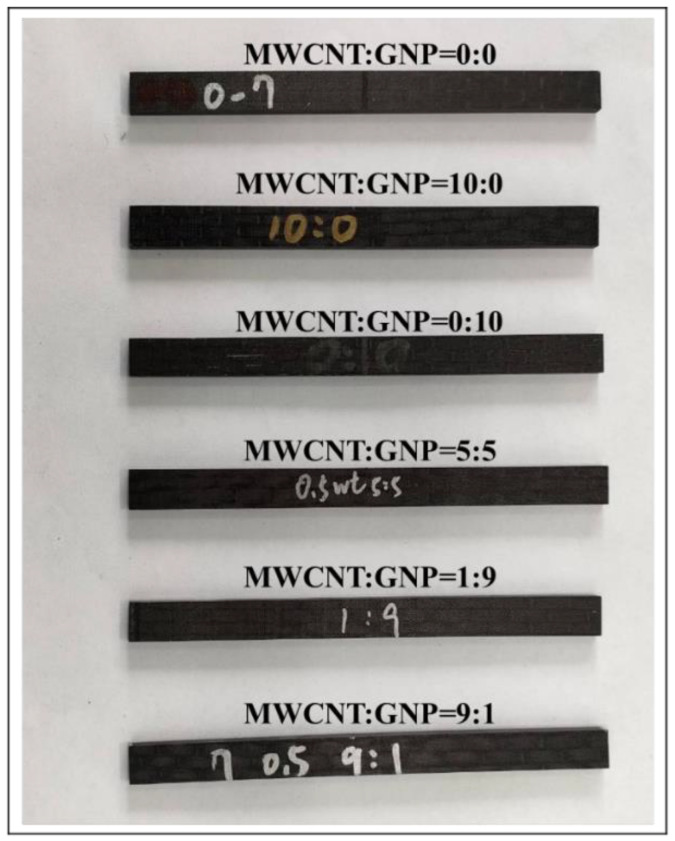
Photograph of all types of Cf/Ep specimens with matrix modified under various nanofiller ratios.

The studied nano-modified Cf/Ep laminate was fabricated using hand lay-up process. The fiber volume fractions of all types of laminate specimens were controlled at 45%. The nano-modified epoxy resin was poured on the fabric with required dimensions and squeegeed using a scraper and a roller to obtain the impregnated fabric layer. Next the fabric was dried at 60 °C to remove the MEK solvent and obtain the impregnated fabric. The laminate was prepared by laying-up 14 layers of impregnated fabrics first according to the ply sequence of [0]_14_. The laid-up laminate was hot-pressed at 150 °C in a vacuum oven. The pressure was increased to 1300 psi gradually and kept constant at 1300 psi for 20 min. Then the specimens with the desired dimensions were obtained by cutting the laminate plate according to the ASTM standard D790-17 [40]. The length *L*_0_, width *w* and thickness *h* of the specimen are 160, 13, and 4 mm, respectively. In the present study, six types of Cf/Ep laminate specimens with matrix modified under various MWCNT:GNP ratios, i.e., 0:0, 10:0, 0:10, 9:1, 5:5, and 1:9, were prepared to study the effect of nanofiller ratio applied in the matrix modification on the flexural monotonic and fatigue properties of Cf/Ep laminates. Note that the specimen with matrix modified under a MWCNT:GNP ratio of 0:0 is the one with neat epoxy matrix. The specimens with matrix reinforced under the MWCNT:GNP ratios of 10:0 and 0:10 represent the ones with matrix modified by MWCNTs only and GNPs only, respectively. The photograph of all types of specimens with matrix modified under various nanofiller ratios is shown in Figure 2.

**Figure 3:**
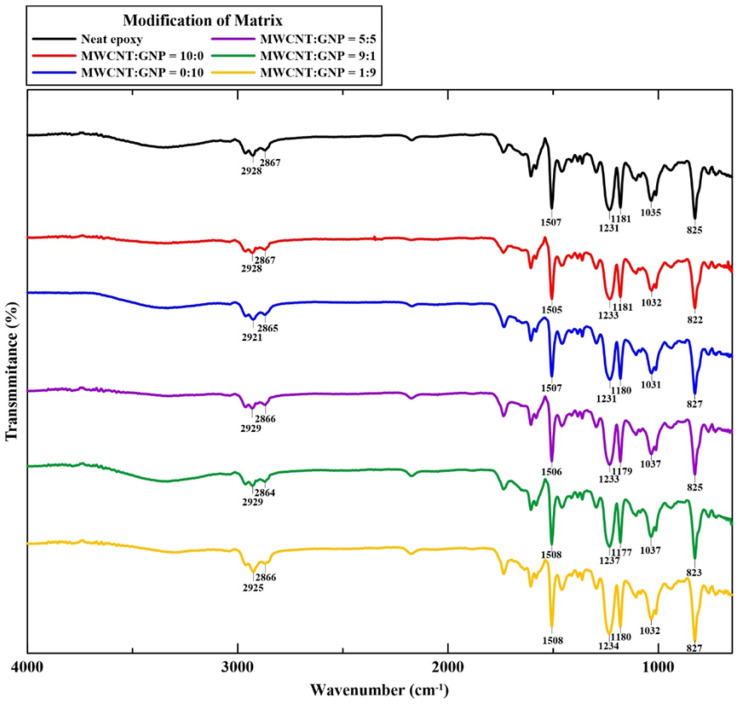
FT-IR spectra for all types of Cf/Ep specimens with matrix modified under different nanofiller ratios.

To understand the chemical composition of the studied Cf/Ep specimens and verify the effect of temperature cycle of specimen preparation on the chemical characterization of the specimens, FT-IR (PerkinElmer, Spectrum Two, Waltham, MA, USA) was performed on all types of specimens with different nanofiller ratios. Figure 3 shows the FT-IR spectra of the studied Cf/Ep laminate specimens with matrix modified under various nanofiller ratios. The variation and characteristic peaks of the FT-IR spectrum curves for all types of specimens are almost identical, revealing that the FT-IR spectrum of the bulk specimen only identifies the characteristic composition of carbon/epoxy composites. Due to the tiny amounts of nanoparticles applied in the modification of laminate specimens, the detailed contributions of nanoparticles cannot be reflected in the spectra. Compared to the FT-IR spectrum of carbon fiber reinforced neat epoxy composites shown in other study [41], the peaks of the studied specimens modified under different nanofiller ratios are almost identical to those of neat Cf/Ep composites. Furthermore, compared to the FT-IR spectrum of neat epoxy [42], Figure 3 also shows that no additional peaks are found for all FT-IR spectrum curves appear, indicating that no oxidation reaction occurred during the temperature cycle of the specimen preparation.

### 2.4. Tests of Mechanical Properties

**Figure 4:**
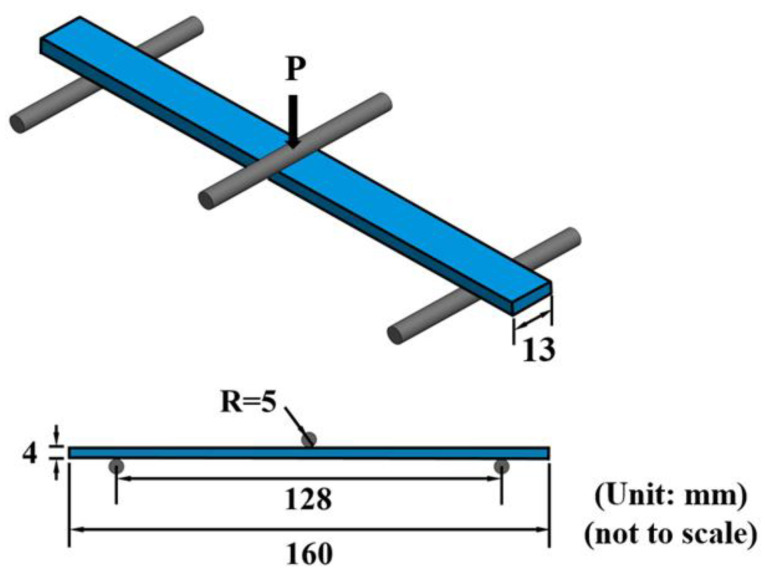
Setup of the three-point flexural test.

The flexural monotonic and fatigue tests were performed using an MTS 810 servo-hydraulic material system (MTS Systems Corporation; Eden Prairie, MN, USA) with a three-pint bending jig. The span between two supporting rollers *L* is 128 mm. The diameter of the loading roller and supporting rollers is 10 mm. The monotonic flexural tests were conducted according to the ASTM standard D790-17 [40]. The setup of the three-point flexural tests is shown in Figure 4. The monotonic tests were performed by controlling the speed of the loading roller at 1 mm/min. The flexural modulus *E_b_* can be obtained using the following equation:

(1) $$E_{b}=\frac{L^{3}m}{4wh^{3}}$$

where *m* is the initial slope of load–displacement curve. The flexural stress *σ_b_* can be obtained using the following equation:

(2) $$\sigma_{b}=\frac{3PL}{2wh^{2}}$$

where *P* is the applied load. The flexural fatigue tests were carried out according to the ASTM standard D7774-17 [43]. The flexural strength *σ_bf_* can be obtained by substituting the peak value of loading *P_c_* in the monotonic flexural test into Equation (2). The fatigue tests were conducted under load-controlled mode. The loading ratio, defined as the ratio of minimum applied load to maximum applied load in one loading cycle, was set to be 0.1. Note that the load or displacement downward is considered as positive. The waveform of the cyclic loading was sinusoidal with a frequency of 3 Hz. For each type of specimens with matrix modified under a specific nanofiller ratio, the fatigue tests were performed under five selective loading levels, i.e., 80, 82.5, 85, 90, and 95%. The loading level is defined as the ratio of the maximum flexural stress in the fatigue test *σ_b,_*_max_ to the monotonic flexural strength *σ_bf_*. The fatigue life is defined as the number of cycles corresponding to the separation of specimen. The fatigue test was interrupted when the applied cycles exceeded one million, and the specimen was considered to have infinite life. Each fatigue test was repeated twice to obtain the reliable experimental data.

After the flexural monotonic and fatigue tests, the fracture surfaces of the specimens were observed using a field emission scanning electron microscope (FE-SEM) (JEOL JSM-7610F, JEOL Ltd., Akishima, Tokyo, Japan) to verify the reinforcement mechanisms of hybrid nanoparticles on the studied mechanical properties of the nano-modified Cf/Ep laminate specimens.

To quantify the synergistic effect of hybrid nanofillers on the mechanical properties, a synergistic index was used to evaluate the synergistic effect of MWCNTs and GNPs on the flexural properties of Cf/Ep laminates. The concept of the utilized synergistic index has been provided in detail in [44,45] and has been applied in the monotonic and cyclic mechanical properties of polymer hybrid nanocomposites. The magnitude of synergistic index depends on the difference between the experimental and predicted properties of the hybrid nano-Cf/Ep laminates. The predicted property is obtained from the properties of the MWCNT-modified specimen and GNP-modified Cf/Ep specimen in accordance with the weight fraction. The synergistic index *η* for any specific mechanical property can be expressed as

(3) $$\eta \left(\%\right)=\frac{\psi^{\left(x:y\right)}-[0.1\cdot (x\cdot \psi^{\left(10:0\right)}+y\cdot \psi^{\left(0:10\right)})]}{0.1\cdot (x\cdot \psi^{\left(10:0\right)}+y\cdot \psi^{\left(0:10\right)})}\cdot 100$$

where $\psi^{\left(x:y\right)}$ is the property of the Cf/Ep laminate specimen with a MWCNT:GNP ratio of *x*:*y* (*x* + *y* = 10); $\psi^{\left(10:0\right)}$ and $\psi^{\left(0:10\right)}$ are the properties of the Cf/Ep specimens with matrix modified by MWCNTs only and GNPs only, respectively. The synergistic effect of hybrid nanoparticles on the studied mechanical property is assessed based on the difference between the actual and predicted magnitudes of the property enhanced by the hybrid nanoparticles. The predicted value of the studied property of the hybrid nano-modified laminates is calculated according to the weight ratio of the properties of the laminate specimens with individual type of nanoparticle. In the composition of predicted property of the Cf/Ep laminate specimen with the matrix modified under a MWCNT:GNP ratio of *x*:*y* (*x* + *y* = 10) the property of the laminate specimen modified by MWCNTs only accounts for *x*/10, and the property of the specimen with the matrix modified by GNPs only accounts for *y*/10. The positive synergistic index demonstrates that the experimental value of the studied property of the hybrid modified Cf/Ep laminates is larger than the predicted one. The larger index represents the stronger synergistic effect of the hybrid nanoparticles on the studied property.

## 3. Results and Discussion

### 3.1. Monotonic Flexural Properties

**Figure 5:**
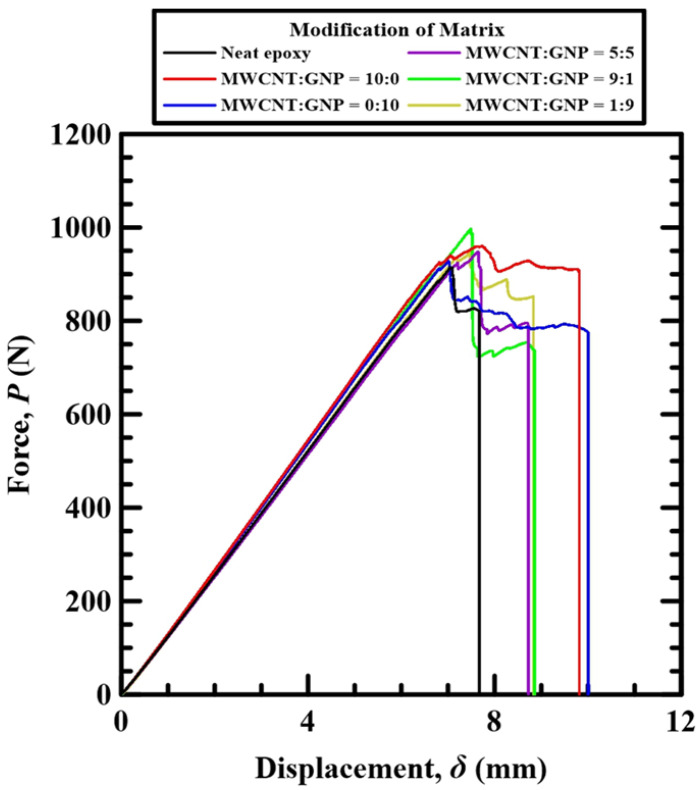
Relationships between the applied loads and the displacements of the loading roller obtained in the monotonic flexural tests.

Figure 5 shows the representative relationships between the applied loads and the displacements of the loading roller obtained in the monotonic flexural tests. It is found that for all Cf/Ep composite specimens with matrix modified under different nanofiller ratios, the load–displacement curves present linear behavior from the beginning of the monotonic tests till the applied loads reach the peak values. Subsequently the applied loads decrease with displacements till the final fracture of specimens.

**Figure 6:**
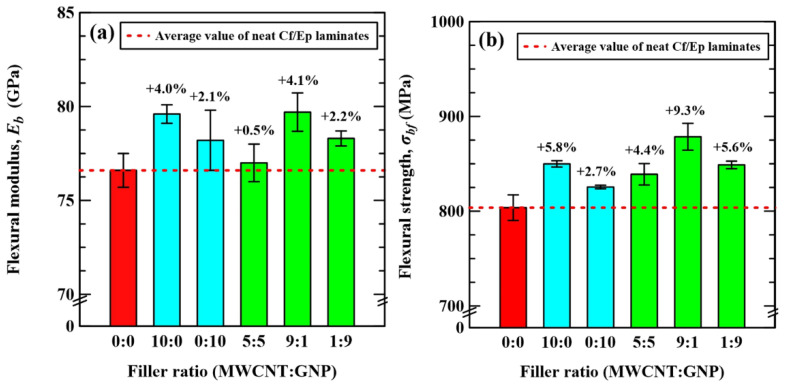
Variations of (**a**) flexural moduli and (**b**) strengths of the studied specimens with the MWCNT:GNP ratios applied in the preparation of matrix modification.

Table 2 lists the experimental data of flexural moduli and strengths of the Cf/Ep laminate specimens with matrix modified under different nanofiller ratios. The variations of flexural moduli and strengths of the studied specimens with the applied nanofiller ratios in the modification of matrix are shown in Figure 6a,b, respectively. Adding individual type of nanofiller in the matrix is found to improve the flexural moduli and strengths of the Cf/Ep laminate specimens. Moreover, the improvement effect on the monotonic properties of MWCNTs is slightly higher than that of GNPs. The synergistic effect of hybrid nanofillers on the monotonic properties of Cf/Ep laminates is found only under special nanofiller ratios. The flexural moduli and strengths of the laminate specimens with matrix reinforced under a MWCNT:GNP ratio of 9:1 are higher than those of neat Cf/Ep laminates by 4.1 and 9.3%, respectively. The monotonic properties of specimens modified under this special nanofiller ratio are also higher than those reinforced by individual type of nanofiller. The stress concentration resulting from the agglomeration of flake-shaped GNPs decreases the monotonic properties of Cf/Ep composites. However, adding MWCNTs between the GNPs can suppress the formation of agglomeration, and provide beneficial bridging network for load transfer, further increase the quasi-static properties.

For the Cf/Ep laminate specimens reinforced by individual type of nanofillers, the MWCNTs display higher improvement on the monotonic flexural properties than GNPs. The π-π interaction and van der Waals force between the graphene layers make the GNPs easy to agglomerate, further reduce the reinforced effect on monotonic flexural properties due to the stress concentration of the nanoparticle agglomeration. The relative concentrations of MWCNTs and GNPs is critical to the monotonic properties of the hybrid nano-modified Cf/Ep laminate specimens. Replacing part of MWCNTs with GNPs can improve the monotonic properties of Cf/Ep composites because the larger surface areas of GNPs provide more crosslink between the nanoparticles and the epoxy matrix. Besides, the MWCNTs can separate the flake-shaped GNPs effectively and constitute beneficial crosslinks between the GNPs for load transfer. However, the agglomeration problem caused by the excessive addition of GNPs is detrimental to the monotonic properties of the Cf/Ep laminates. The explanations described above clarify the flexural modulus and strength of the Cf/Ep laminate specimens modified under the MWCNT:GNP ratio of 9:1 is higher than those modified under the nanofiller ratio of 1:9.

**Figure 7:**
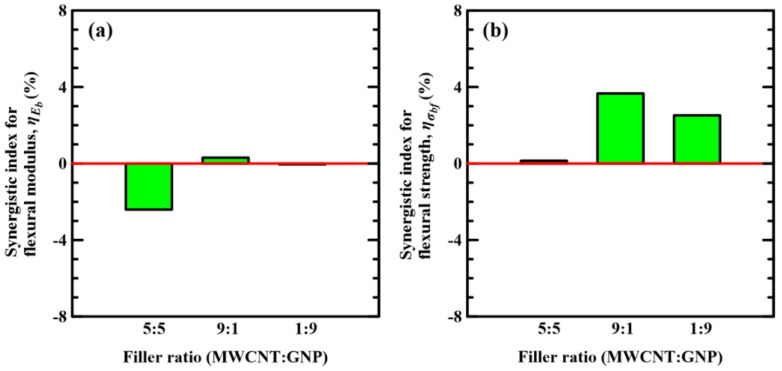
Variations of synergistic index of (**a**) flexural moduli and (**b**) flexural strengths with the applied nanofiller ratios of hybrid-nano-Cf/Ep laminates.

Figure 7 shows the variations of synergistic indices of flexural moduli and flexural strengths with the applied nanofiller ratios of hybrid-nano-Cf/Ep laminates. It is evident that the specimens with matrix reinforced under the MWCNT:GNP ratios of 9:1 and 1:9 present significant synergistic effect of hybrid nanofillers, and the ones with matrix modified under the MWCNT:GNP ratio of 5:5 display slight or reversed influence on the studied flexural properties. The results shown in Figure 7 indicate that the applied synergistic index is an effective tool with high degree of discrimination for evaluating the benefits of hybrid nanofillers on the studied mechanical properties. The specimens with matrix modified under a MWCNT:GNP of 5:5 display negative synergistic index for flexural modulus and approaching zero index for flexural strength, indicating the synergistic effect of the hybrid nanofillers on under this specific nanofiller ratio have effect on the improvement of flexural modulus. The specimens with matrix modified under the nanofiller ratios of 1:9 and 9:1 display tiny synergistic indices for the flexural modulus, revealing that although the moduli of the specimens modified under the nanofiller ratios of 1: and 9:1 are higher than that of the neat laminate specimen, but lower than the predicted value estimated by the weight ratio of properties of the specimens modified with individual type of nanofiller. Furthermore, the specimens with 1:9 and 9:1 nanofiller ratios present positive synergistic indices for the flexural strength, elucidating that synergistic effect of the hybrid nanofillers on the strength is significant. The fact that the flexural strength of the specimens with a 9:1 nanofiller ratio is higher than that of the specimens with a 1:9 nanofiller ratio is also reflected in the corresponding indices of these two types of specimens.

**Figure 8:**
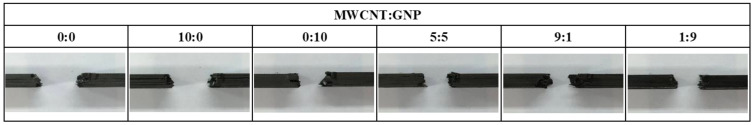
Edge views of the static-failed Cf/Ep specimens with matrix modified under different nanofiller ratios.

Figure 8 shows the edge views of the static-failed specimens with matrix modified under different nanofiller ratios. The locations of fracture for all type of specimens modified under various nanofiller ratios are below the loading roller. The fiber breakage and delamination are the main failure modes observed on the failed specimens obtained after the monotonic flexural tests.

### 3.2. Fatigue Flexural Properties

**Figure 9:**
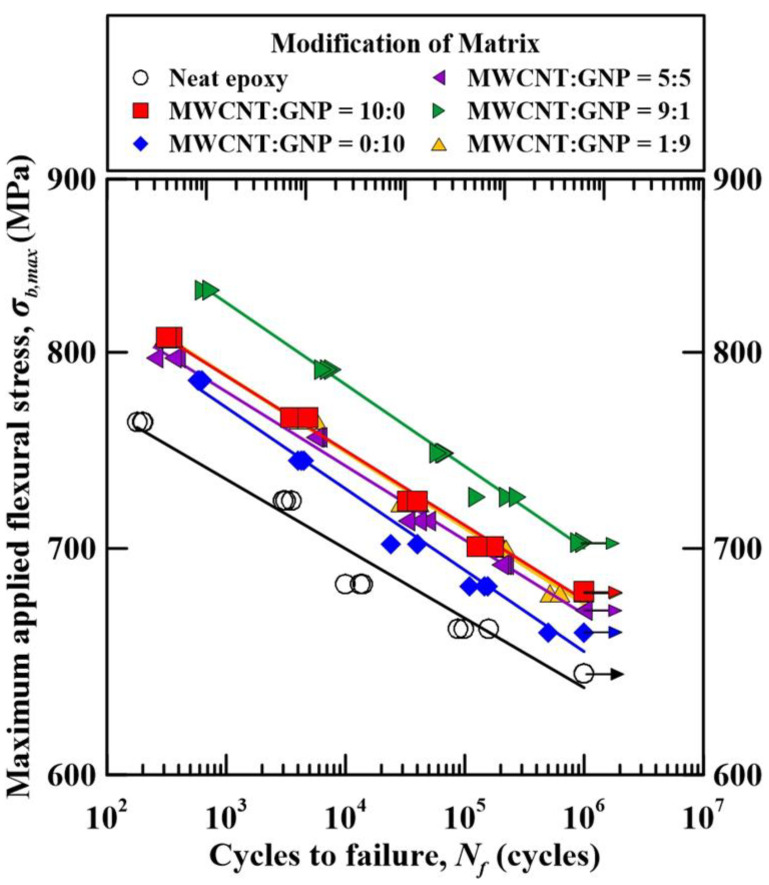
Relationship between the maximum applied flexural stress and the fatigue lives for the Cf/Ep laminate specimens with matrix modified under various nanofiller ratios.

The experimental results obtained from the fatigue tests of the studied Cf/Ep composite specimens with matrix reinforced under different nanofiller ratios are listed in Table 3. Figure 9 shows the relationships between the maximum applied flexural stresses *σ_b,_*_max_ and the fatigue lives *N_f_* for the specimens with matrix modified under various nanofiller ratios. The arrows shown in this figure represent the specimens survived after 10^6^ cycles. For each type of specimen with matrix modified under a specific nanofiller ratio, a power-law model was employed to describe the relationship between the applied stresses and fatigue lives, which can be expressed as

(4) $$\sigma_{b,\max}=AN_{f}{}^{B}$$

where *A* is the fatigue strength coefficient, and *B* is the fatigue strength exponent. These two material constants can be obtained by fitting the experimental data with the proposed model. The fitting results of the two material constants are also listed in Table 3. The coefficients of determination *R*^2^ were used to evaluate the fitting results of the proposed model. All the values of *R*^2^ for the specimens with different nanofiller ratios are larger than 0.95, demonstrating that the power-law model is appropriate to describe the stress-life relationship. The obtained power-law models for all types of specimens modified under various nanofiller ratios are also plotted in Figure 9, which present as linear lines in the log-log scale diagram.

Similar to the monotonic behavior, the fatigue strength of MWCNT-modified Cf/Ep laminates is higher than that of GNP-modified Cf/Ep laminates. The bridging and pullout effects of MWCNTs suppress the fatigue crack propagation rate significantly. In spite that the crack deflection effect caused by GNPs retards the crack growth, the stress concentration resulting from the GNP agglomeration accelerates the crack initiation at the early stage of the fatigue tests. The poor dispersion of GNPs also explains the fatigue strength of the specimens modified under nanofiller ratio of 1:9 is lower than that modified under the nanofiller ratio of 9:1.

**Figure 10:**
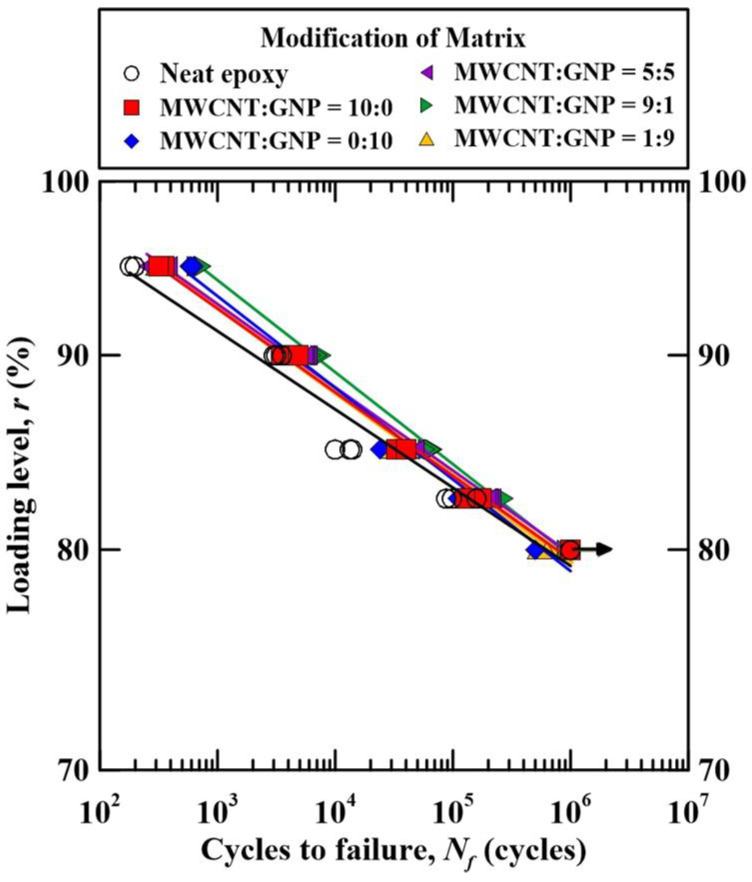
Relationship between the applied loading levels and the fatigue lives for the Cf/Ep laminate specimens with matrix modified under various nanofiller ratios.

Figure 10 shows the relationships between the applied loading levels and the fatigue lives of the studied Cf/Ep laminate specimens with matrix modified under different nanofiller ratios. This figure is obtained by replacing the vertical axis of Figure 9 from the maximum applied stress to the applied loading levels. It is seen that the loading level-life curves of the laminate specimens with matrix modified under different nanofiller ratios almost coincide together. The identical slopes of all curves demonstrate that the applied loading levels of all types of specimens reinforced under different nanofiller ratios have the same sensitivity to the fatigue lives. For all types of laminate specimens modified under various nanofiller ratios, the loading levels for the fatigue lives of 103 and 106 are approximately 90% and 80%, respectively.

**Figure 11:**
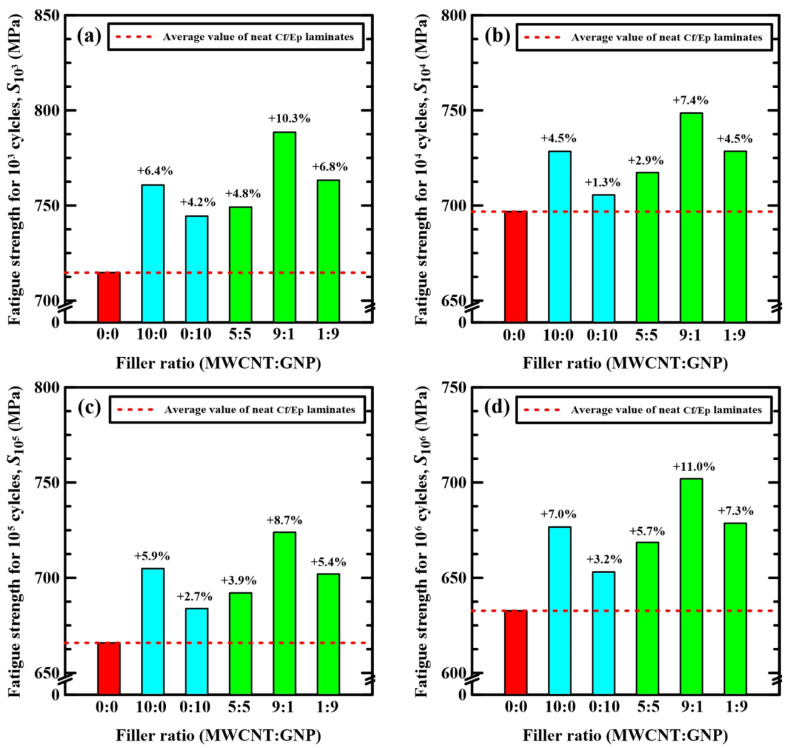
Variation of fatigue strengths at (**a**) 10^3^, (**b**) 10^4^, (**c**) 10^5^, and (**d**) 10^6^ cycles with the nanofiller ratios applied in the matrix modification of the studied Cf/Ep laminate specimens.

To further analyze the influence of hybrid-nanofillers on the fatigue behavior, the variations of fatigue strengths at 10^3^, 10^4^, 10^5^, and 10^6^ cycles (*S*_10_^3^, *S*_10_^4^, *S*_10_^5^, and *S*_10_^6^) for the studied Cf/Ep laminate specimens with the applied nanofiller ratios in the matrix modification are shown in Figure 11a–d, respectively. Note that the fatigue strengths at 10^6^ cycles, S_10_^6^, can be considered as the pseudo endurance limits of the studied nano-Cf/Ep laminates. Figure 11 shows that adding individual type of nanofiller in the matrix can increase the fatigue strength of neat-Cf/Ep laminates. Similar to the monotonic behavior, using MWCNTs as the reinforcement particles is superior to GNPs in improving the fatigue strength. For the hybrid nano-Cf/Ep laminates, only the specimens with matrix modified under a MWCNT:GNP ratio of 9:1 have higher fatigue strength than the ones with matrix modified by MWCNTs only and GNP only. The fatigue strength of the Cf/Ep composite specimens with matrix modified under the MWCNT:GNP ratio of 5:5 and 1:9 is higher than that of the specimens modified under a MWCNT:GNP ratio of 0:10, but lower than that of the specimens modified under a MWCNT:GNP ratio of 10:0.

**Figure 12:**
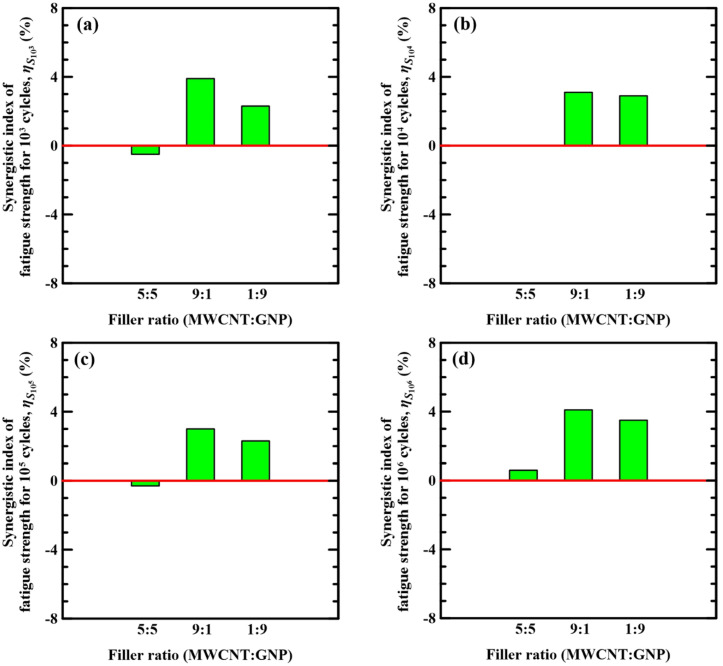
Variations of synergistic indices of the fatigue strength at (**a**) 10^3^, (**b**) 10^4^, (**c**) 10^5^, and (**d**) 10^6^ cycles with the nanofiller ratios applied in the matrix modification of the studied Cf/Ep laminate specimens.

Figure 12a–d show the variation of synergistic indices for the fatigue strength at 10^3^, 10^4^, 10^5^, and 10^6^ cycles, i.e., *S*_10_^3^, *S*_10_^4^, *S*_10_^5^, and *S*_10_^6^, with the nanofiller ratios applied in the matrix modification of the studied Cf/Ep laminate specimens, respectively. It is evident that the hybrid nano-Cf/Ep composites with matrix modified under MWCNT:GNP ratios of 9:1 and 1:9 have positive synergistic indices of the fatigue strength. Oppositely, the synergistic indices for the fatigue strengths of the Cf/Ep composites modified under the MWCNT:GNP ratio of 5:5 are either slightly positive or negative, implying that the synergistic effect of hybrid nanofillers on the fatigue strength of the specimens with matrix modified under the MWCNT:GNP ratio of 5:5 is negligible.

For the specimens with a 5:5 nanofiller ratio, all the synergistic indices for the fatigue strength at various life levels are approaching zero, indicating that the synergistic effect of hybrid nanofillers under a 5:5 ratio on the fatigue strength is weak. Oppositely, the specimens with 9:1 and 1:9 nanofiller ratios present positive synergistic indices for the fatigue strength at various life levels, demonstrating that the hybrid nanofillers under these disparate ratios have remarkable synergistic effect on the fatigue strength of the Cf/Ep laminates. Furthermore, the fact that the specimens with a 9:1 nanofiller ratio is better than the ones with a 1:9 nanofiller ratio in the performance of fatigue resistance can also be highlighted from the comparison of the corresponding indices.

**Figure 13:**
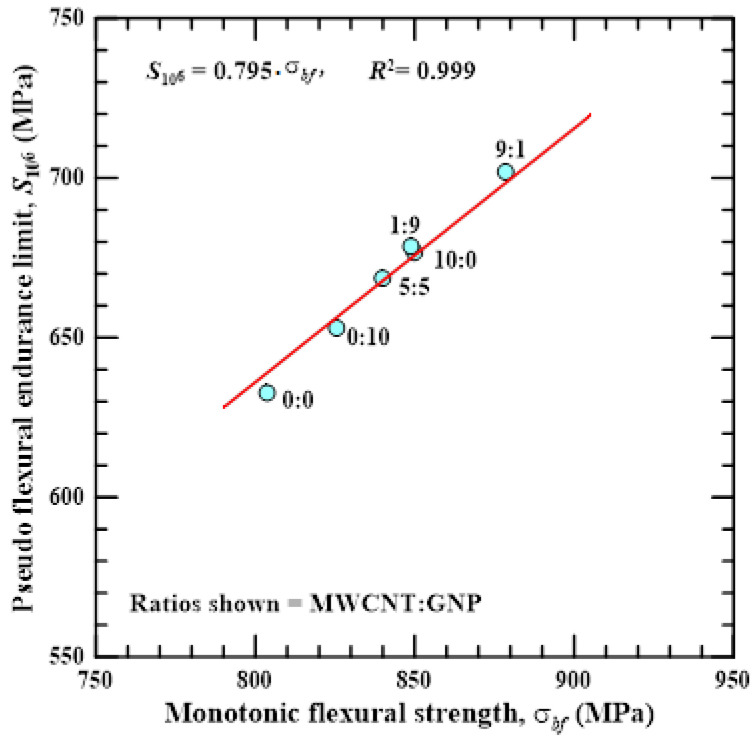
Relationship between the pseudo endurance limits and monotonic flexural strengths of the Cf/Ep laminates with matrix modified under various nanofiller ratios.

In the fatigue design of engineering materials, knowing the relationship between the quasi-static and fatigue strengths is critical in the selecting of materials for practical application. Figure 13 shows the relationship between the monotonic flexural strengths *σ_bf_* and the pseudo endurance limits *S*_10_^6^ of the Cf/Ep laminates with matrix modified under different nanofiller ratios. This figure demonstrates that the fatigue strength of the studied Cf/Ep laminates increases with the monotonic strength, no matter what nanofiller ratio is applied in the matrix modification. An empirical relationship can be applied to describe the relationship between fatigue and monotonic flexural strengths, which can be expressed as a linear equation:

(5) $$S_{10^{6}}=0.795\cdot \sigma_{bf}$$

The coefficient of determination *R*^2^ of the fitting results for Equation (5) is as high as 0.999, indicating that the flexural fatigue limit of the studied nano-Cf/Ep laminates can be determined as 0.8 times the monotonic strength. This empirical equation provides a quick and convenient guideline for selecting of nano-modified Cf/Ep composites under anti-fatigue considerations.

**Figure 14:**
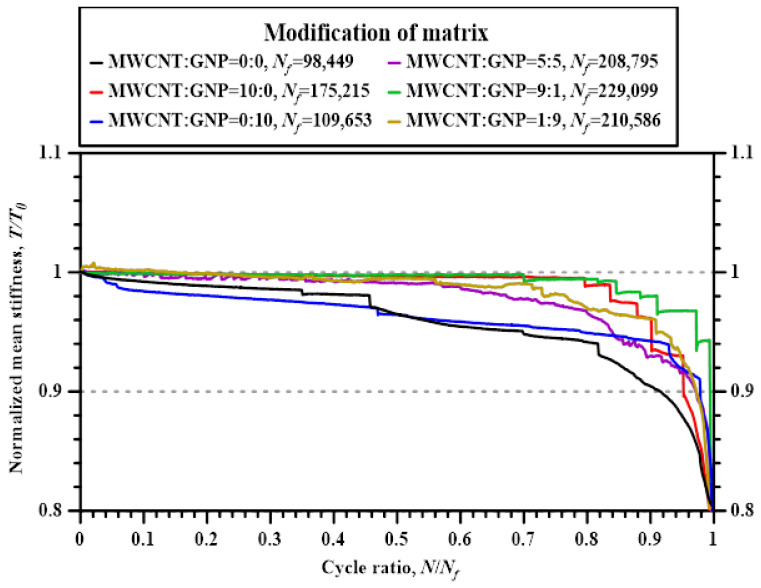
Variations of normalized stiffness with the applied cycle ratios for the Cf/Ep laminate specimens with matrix modified under various nanofiller ratios.

Figure 14 displays the variations of normalized stiffness with the applied cycle ratios for the Cf/Ep laminate specimens with matrix modified under various nanofiller ratios. The stiffness *T* is defined as the ratio of applied load range Δ*P* to the displacement range Δ*δ*, and the normalized quantity is obtained from dividing the stiffness of *n*th cycle by the initial stiffness of specimen *T*_0_. For comparative purpose, the representative experimental data shown in this figure are selected from the specimens with fatigue lives ranged from 100,000 to 250,000 cycles. It is obvious that the stiffness of neat-Cf/Ep laminate specimen and the specimen modified by GNPs only decreases continuously from the beginning of the fatigue test to the final fracture. Furthermore, the stiffness of neat Cf/Ep laminates decreases rapidly in the last 20% of lifespan. Using MWCNTs instead of GNPs as the reinforcement particles can retard the stiffness degradation of the Cf/Ep laminates, the stiffness remains almost constant till 80% of the lifespan. Besides, dispersing hybrid nanoparticles in the matrix can suppress the stiffness degradation of the studied Cf/Ep laminate specimens. The stiffness of the specimens with matrix modified under the MWCNT:GNP ratios of 1:9 and 5:5 does not decrease till half of lifespan. Moreover, the stiffness of the Cf/Ep laminate specimen with matrix modified under a MWCNT:GNP ratio of 9:1 remain unchanged till 80% of lifespan, indicating adding hybrid nanoparticles under appropriate allocation ratios in the matrix can retard the degradation of stiffness effectively.

**Figure 15:**
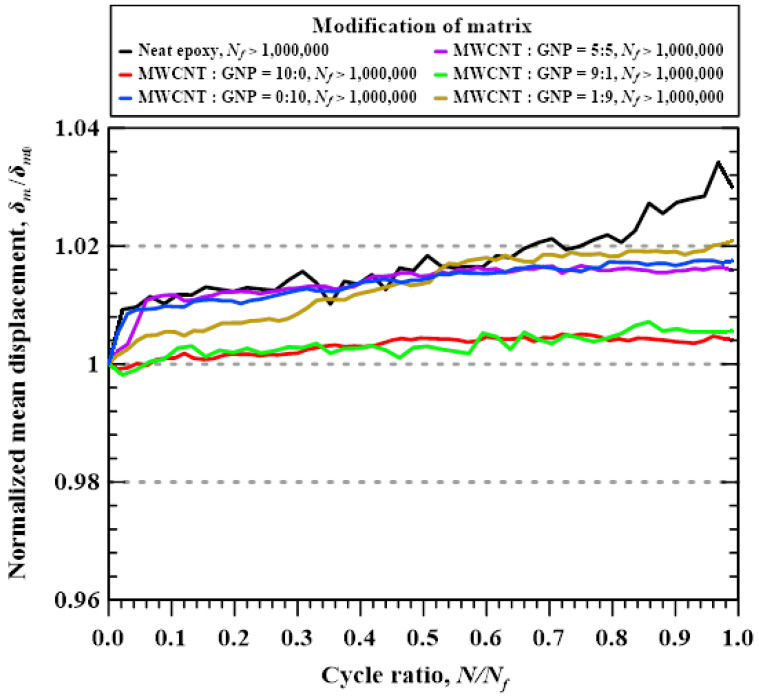
Representative variations of normalized mean displacements with the applied cycle ratios for the studied Cf/Ep laminate specimens with matrix modified under various nanofiller ratios.

Figure 15 shows the representative variations of normalized mean displacements with the applied cycle ratios for the studied Cf/Ep laminate specimens with matrix modified under various nanofiller ratios. Here the normalized mean displacement is defined as the mean displacement in the *n*th cycle *ε_m_* divided by the initial mean displacement *ε_m_*_0_. The experimental results shown in the figure is obtained from the representative specimens with fatigue lives larger than 10^6^ cycles. The mean displacement is an indicator of dynamic creep behavior in the load-controlled fatigue tests. In general, the mean displacement or strain increases with the applied cycles when the dynamic creep behavior of the tested material is significant. The phenomenon of dynamic creep is frequently observed on the ductile materials or the materials experiencing plastic deformation. It is shown that the normalized mean displacements of the specimens with matrix modified under the MWCNT:GNP ratios of 0:0, 0:10, and 5:5 increase rapidly at the beginning stage of the fatigue tests, and the mean displacements continue to increase with the applied cycles till the final stage. It demonstrates that the Cf/Ep laminates with matrix modified under these three nanofiller ratios display significant dynamic creep behavior. Moreover, the mean displacement of the specimens with matrix modified under a MWCNT:GNP ratio of 1:9 increase gradually with the applied cycles in the whole lifetime. The normalized mean displacements of the laminate specimens with matrix modified under the MWCNT:GNP ratios of 10:0 and 9:1 increase slightly with the applied cycles, demonstrating that the dynamic creep behavior for the specimens with matrix reinforced under these two nanofiller ratios is insignificant. The trend that the dynamic creep behavior varies with the applied nanofiller ratios is opposite to that the tensile strength changes with the employed nanofiller ratios. The studied nano-Cf/Ep laminate specimen with higher tensile strength displays less significant dynamic creep characteristics.

**Figure 16:**
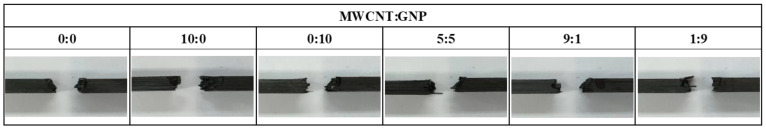
Edge views of the fatigue-failed Cf/Ep specimens with matrix modified under different nanofiller ratios.

The edge views of the fatigue-failed laminate specimens with matrix modified under different nanofiller ratios are presented in Figure 16. Similar to the static-failed specimens, the fiber fracture and delamination are the most dominant failure modes observed after the fatigue tests. Furthermore, the fatigue-failed specimens display more delamination than the static-failed ones, indicating that the interlaminar properties plays an important role in the flexural fatigue strength of the Cf/Ep specimens.

### 3.3. Observation of Fracture Surfaces

**Figure 17:**
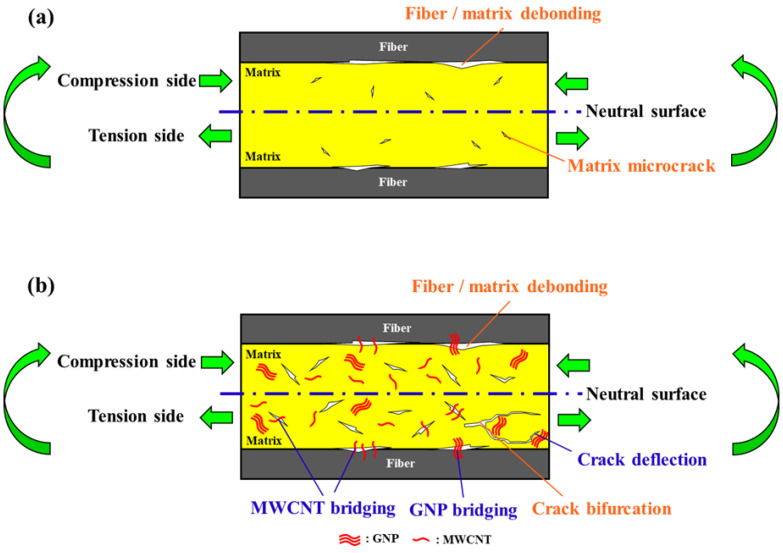
Schematic illustration of (**a**) fracture mechanisms of the neat Cf/Ep laminates subjected to cyclic flexural loading; (**b**) reinforcement mechanisms provided by the nanoparticles on the fatigue flexural strength of the hybrid nano-Cf/Ep laminates.

**Figure 18:**
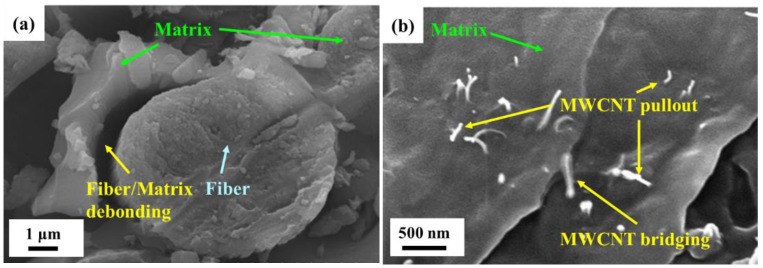

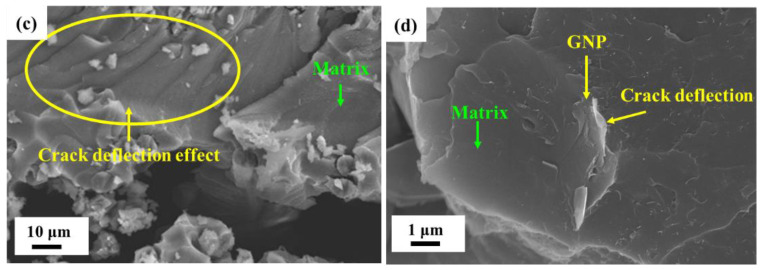
(**a**) Fiber/matrix debonding observed on the fracture surface of the laminate specimen with neat epoxy matrix; (**b**) pullout and bridging effects of MWCNTs observed on the fracture surface of the laminate specimen with matrix modified under a MWCNT:GNP ratio of 5:5; (**c**) ripple-like fracture surface of the laminate specimen with matrix modified under a MWCNT:GNP ratio of 1:9; (**d**) crack deflection observed on the fracture surface of the laminate specimen with matrix modified under a MWCNT:GNP of 9:1.

Figure 17a depicts the schematic illustration of fracture mechanisms of the studied Cf/Ep laminates with neat epoxy matrix subjected to cyclic flexural loading. Owing to the characteristics of bending loads, the difference of fracture mechanisms between the tension and compression sides of the studied neat Cf/Ep laminate specimen can be observed. On the tension side, the multiple microcracks form in the matrix at the beginning stage of the fatigue test. Then the microcracks grow and link together with the applied tensile cycles, and some main cracks propagate to the fiber/matrix interfaces to develop the debonding damage. The interfacial debonding continues to evolve into the delamination, and the delamination propagates till the final fracture. Different from the damage development observed on the tension side, the fiber/matrix debonding due to the buckling of fibers dominates the fatigue damage on the compression side at the beginning stage of fatigue test instead of the formation and linkage of microcracks. The debonding develops into delamination rapidly till the final fracture. Figure 18a shows the SEM image of the fracture surface of neat Cf/Ep laminate specimen obtained in the fatigue test. The fiber/matrix debonding can be observed clearly on the compression side of the fracture surface.

The reinforcement mechanisms provided by MWCNTs and GNPs are illustrated in Figure 17b. The pullout and bridging effects of MWCNTs observed in the matrix improve the fatigue strength by suppressing the initiation and growth of microcracks on the tension side. Figure 18b shows the SEM image of pullout and bridging of MWCNTs in the matrix of the Cf/Ep laminate specimen with a MWCNT:GNP ratio of 5:5. Moreover, the bridging effect of MWCNTs at the fiber/matrix interfaces enhances the resistance to debonding and delamination, further improves the fatigue strength. The crack bifurcation and crack deflection effect are the main reinforcement mechanisms provided by the two-dimensional flake-shaped GNPs. The cracks need more energy consumed to bifurcate or change the direction of propagation when encounter the GNP particles. These reinforcement mechanisms of GNPs result in the wavy fracture surface. Figure 18c shows the fracture surface of fatigued Cf/Ep laminate specimen with matrix modified under a MWCNT:GNP of 1:9. The ripple appearance of fracture surface verifies the toughening mechanism of crack deflection effect of GNPs. The enlarged SEM image of the fracture surface obtained from the laminate specimen with matrix modified under a MWCNT:GNP ratio of 9:1 is shown in Figure 18d, the matrix surfaces on both sides of the GNP particles present different heights, indicating the crack front changes the direction of propagation to bypass the GNP particles. Furthermore, the bridging effect of GNPs at the fiber/matrix interfaces provides secondary reinforcement mechanism to retard the development of debonding and delamination. The synergistic effect of MWCNT-GNP hybrids can be attributed to the excellent load transfer provided by the MWCNT networks located between the GNPs. Moreover, adding MWCNTs in the GNP-modified matrix can prevent the agglomeration of GNPs, further enhance fatigue strength of Cf/Ep laminates by eliminating the stress concentrations [46,47].

## 4. Conclusions

This work studied the influence of mixing MWCNTs and GNPs in the matrix on the monotonic and fatigue strengths of Cf/Ep laminate specimens. The static and fatigue properties of the Cf/Ep laminates with matrix modified under various nanofiller ratios have been experimentally obtained and analyzed. The synergistic effect of hybrid nanoparticles has been evaluated using a synergistic index. The reinforcement mechanisms of the nanoparticles have been verified by observing the fracture surfaces of the specimens. Some conclusions can be summarized as

1. Adding individual type of nanofiller can improve the monotonic properties of the Cf/Ep laminates. The laminate specimen with matrix modified under a MWCNT:GNP ratio of 9:1 displays higher flexural modulus and strength than the ones with individual type of nanofiller and the hybrid ones with other nanofiller ratios. MWCNT
2. The MWCNTs present better performance in improving the fatigue strength of Cf/Ep laminates than the GNPs. The hybrid nano-Cf/Ep laminates with matrix modified under a MWCNT:GNP ratio of 9:1 demonstrates better synergistic effect on the fatigue strength than those modified under other nanofiller ratios.
3. The addition of small amounts of GNPs in the MWCNT-modified polymer matrix can provide larger surface areas for crosslink between the nanoparticles and the polymer matrix. Moreover, the MWCNTs can separate GNPs effectively to avoid the agglomeration of GNPs and constitute the networks between the GNPs for load transfer, further improve the monotonic and fatigue properties of the Cf/Ep laminates.
4. The utilized synergistic index is a tool with high discrimination to evaluate the synergistic effect of hybrid nanoparticles on the studied mechanical properties.
5. The flexural endurance limits of the studied nano-modified Cf/Ep laminates increase with the monotonic strengths.
6. The pullout effect of MWCNTs and the crack deflection effect of GNPs are the main reinforcement mechanisms to improve the fatigue strength of the Cf/Ep laminates. The bridging effects of the nanoparticles at the matrix crack sites and fiber/matrix interfaces provide another toughening mechanism to resist the damage development.

## Figures and Tables

**Table 1:** Specifications of the materials used in this study.

Reinforcement	Matrix	Nanofillers	
Carbon Fabric	Epoxy	MWCNT	GNP
Fiber count in tow: 12 K Areal weight: 216 g/m^2^ Thickness: 0.28 mm	Bisphenol A type Epoxy resin: dicyanamide curing agent = 18:2 Solid content: 60% Curing temperature: 125–150 °C Viscosity: 24 mpa·s	Diameter: ~9.5 nm Length: ~1.5 μm Purity: >90%	Diameter: ~5 µm Thickness: ~10 nm Purity: >99.5%

**Table 2:** Experimental results of the flexural monotonic tests for the Cf/Ep laminate specimens with matrix modified under various MWCNT:GNP ratios.

Nanofiller Ratios MWCNT:GNP	Flexural Modulus *E_b_* (GPa)	Flexural Strength *σ_bf_* (MPa)
0:0	76.57 ± 0.85	803.7 ± 13.5
10:0	79.64 ± 0.46	849.9 ± 3.3
0:10	78.16 ± 1.61	825.5 ± 1.9
5:5	76.98 ± 1.05	839.9 ± 11.3
9:1	79.70 ± 0.96	878.5 ± 14.2
1:9	78.29 ± 0.40	848.8 ± 4.0

**Table 3:** Experimental results of the flexural fatigue tests for the Cf/Ep laminate specimens with matrix modified under various MWCNT:GNP ratios.

Nanofiller Ratio MWCNT:GNP	Loading Level *r* (%)	Fatigue Life *N_f_* (Cycles)	Fatigue Strength Coefficient *A*	Fatigue Strength Exponent *B*	Coefficient of Determination *R^2^*
0:0	80	>1,000,000, >1,000,000, >1,000,000	845.6	−0.021	0.96
	82.5	98,449, 157,430, 87,937			
	85	13,858, 10,041, 13,245			
	90	3002, 3152, 3548			
	95	200, 179, 197			
10:0	80	>1,000,000, >1,000,000, >1,000,000	917.0	−0.022	0.99
	82.5	156,454, 175,215, 125,986			
	85	35,017, 33,285, 40,137			
	90	4863, 3468, 4862			
	95	313, 354, 314			
0:10	80	1,000,000, 1,000,000, 500,484	909.8	−0.024	0.98
	82.5	156,606, 109,653, 146,599			
	85	39,818, 23,845, 40,339			
	90	4034, 4269, 4475			
	95	622, 580, 600			
5:5	80	>1,000,000, >1,000,000, >1,000,000	906.1	−0.022	0.99
	82.5	218,020, 208,795, 197,017			
	85	48,388, 40,896, 32,602			
	90	6015, 5683, 5503			
	95	390, 250, 360			
9:1	80	>1,000,000, >1,000,000, 899,403	977.9	−0.024	0.99
	82.5	229,099, 275,826, 127,150			
	85	68,521, 66,892, 60,159			
	90	7848, 7260, 6432			
	95	752, 646, 751			
1:9	80	518,803, >1,000,000, 625,264	919.6	−0.022	0.99
	82.5	213,637, 210,586, 220,651			
	85	39,594, 28,953, 41,634			
	90	5680, 4064, 4752			
	95	295, 362, 301			

## Data Availability

Not applicable.
